# A tool to evaluate the integration of implementation science methods in a research project: The Dissemination and Implementation Research Capability Self Survey (DIRC-SS)

**DOI:** 10.1017/cts.2026.10711

**Published:** 2026-02-13

**Authors:** Heather J. Gotham, Briana M. Patrick, Praise F. Olatunde, Jane P. Kim, Helene Chokron Garneau, Sara J. Becker, C. Hendricks Brown, William C. Becker, Mark P. McGovern

**Affiliations:** 1 Psychiatry and Behavioral Sciences, Stanford University School of Medicine, USA; 2 Northwestern University Institute for Public Health and Medicine, USA; 3 Northwestern University Feinberg School of Medicine, USA; 4 Yale School of Medicine, USA

**Keywords:** Implementation science, research design, psychometrics, CTSA, methods

## Abstract

**Introduction:**

Clinical and Translational Science Awards (CTSAs) are positioned to enhance the integration of rigorous implementation research methods into projects across their networks, but lack a systematic, standardized process to do so. This study introduces the Dissemination and Implementation Research Capability Self Survey (DIRC-SS), a pragmatic instrument to evaluate and integrate implementation science methods in traditional research activities.

**Methods:**

We developed the 15-item DIRC-SS to assess researchers’ use of implementation research methods across five key constructs. Its reliability (inter-rater agreement and internal consistency) and sensitivity (change over time) were examined in 10 NIH-funded research projects via ratings assigned by the research teams and by implementation science experts at baseline and one year later.

**Results:**

The DIRC-SS total score demonstrated good internal consistency and inter-rater reliability increased over one year. Although the research team ratings did not change significantly over time, the expert ratings significantly increased, and effect sizes across research teams and expert raters were large in this small sample study.

**Conclusions:**

The DIRC-SS demonstrated good internal consistency reliability and moderate inter-rater reliability. It effectively distinguished between different levels of implementation research methods integration. Unlike tools focused on grant proposals or final reports, the DIRC-SS can be used at any point in the research process by a research team as a self-survey, by implementation science experts in a consultation process, or across a CTSA program to characterize the implementation science methods employed across projects and highlight targeted areas for researcher education and training.

## Introduction

Throughout healthcare, discoveries of effective prevention, early intervention, and treatment services often are not translated into routine practice. This results in major gaps in patient access to the best possible interventions. Implementation science is the study of how to move effective interventions into practice, considering: contextual factors that increase or decrease the likelihood of uptake, strategies to help users put interventions in place, and measures of how much and how well an intervention is delivered [1,2]. Although the bulk of implementation research is conducted in the Clinical Implementation stage, there is increasing recognition of its value within the Clinical Research stage [3]. Moving implementation science upstream into the Clinical Research stage can help clinical trialists and intervention developers to understand the potential and eventual real world “implementability” of their innovative interventions (e.g., drugs, devices, team-based procedures, care delivery) and increase the potential for implementation and true public health impact [4]. Clinical and Translational Science Award (CTSA) hubs are now required to include implementation science activities as part of their efforts to advance translational science [5,6].

Institutional CTSAs funded by the National Center for Advancing Translational Science have a range of structures and processes to support the use of implementation research methods [6]. Implementation science expertise may be situated in cores, groups, consortiums, and networks, or within institutional or externally funded (e.g., National Institutes of Health [NIH]) centers. Across these structures, the process of acquiring implementation research support typically involves a researcher or team sharing a draft of their proposed specific aims page or an outline of the study protocol with implementation experts. The type of implementation research support ranges from training, to consultation (e.g., selecting implementation frameworks, strategies, measures), to an expert serving as the “card-carrying implementation scientist” on grant applications.

Although this approach is common, it lacks specification and generalizability [7]. Also, how the expert is evaluating the research project is less than transparent. Lastly, the ironic complexity of implementation science theories, models, and frameworks leaves most researchers beleaguered. In other words, implementation science has a translational problem of its own [8]. We posit that a more standardized and transparent approach to assessing the implementation research methods of a project would have enormous utility for individual researchers, CTSA hubs, and the field at large.

Many instruments, scales, and tools have been developed to measure implementation science constructs and domains such as barriers and facilitators to adoption (over 400 by 2015 [9] with countless others in the last 10 years); however, very few assess the extent to which implementation research methods are part of the research process. The ImplemeNtation and Improvement Science Proposals Evaluation CriTeria (INSPECT) tool [10] was designed for grant reviewers to assess 10 constructs considered to be key ingredients of high-quality implementation research proposals [11]. The INSPECT criteria help reviewers consider the quality of implementation and improvement sciences in the proposed implementation research study and identify a score for each construct. Another tool, the Standards for Reporting Implementation Studies (StaRI) checklist, provides guidelines to improve consistency in the description of implementation studies [12]. The 27-item checklist is specifically for studies assessing the effects of implementation strategies, and prompts implementation researchers to describe both the implementation strategies that support the intervention and health outcomes in the target population.

While both the INSPECT and StaRI assist implementation researchers to assess the implementability of their studies, their applicability is limited to the initiation and reporting stages, they do not focus on all of the core implementation research constructs, and they are designed with a level of detail best suited for implementation science experts. Regarding timing, the INSPECT tool is specifically for implementation research projects at the grant application stage (e.g., setting readiness, feasibility of proposed research, care/quality gap), while the StaRI is used when reporting findings at the end of the study (e.g., do the title and abstract note that it is an implementation study). Regarding coverage, both tools lack key implementation research constructs. For instance, StARI does not include partner engagement, and INSPECT does not include contextual determinants. Extant tools also require a sophisticated level of understanding of complex implementation research methods. Thus, there is a need for an off-the-shelf tool that is accessible for non-implementation science experts and useful for assessing implementation capability throughout the research process as changes are made to bring implementation methods into the project.

Herein, we describe the development and initial use of a pragmatic and streamlined measure for non-implementation science experts to self-assess the degree to which a research project incorporates implementation methods: the Dissemination and Implementation Research Capability Self Survey (DIRC-SS). The aims of this study are to describe the DIRC-SS, assess its reliability, and describe the change in DIRC-SS scores across a sample of 10 research projects from baseline to one-year follow-up.

## Materials and methods

### Scale development

The research team, composed of six implementation science experts from various institutions, conducted a literature review to identify frequently used implementation research dimensions and existing measures of how well projects integrate implementation research methods. The group pinpointed five key components as central to implementation-informed research, aligned with the Implementation Research Logic Model [13], and developed an initial set of items:

- *Evidence for the intervention* focuses on the extent to which the intervention has proven efficacy and effectiveness backed by scientific literature. The level of evidentiary support for the intervention guides the selection of an appropriate study design.
- *Partner engagement* refers to how much research teams engage representative communities and constituents in the research process. Partners from the intervention site and community, especially those with lived experiences and persons from historically marginalized groups, have an essential role in shaping the effective delivery of the intervention by defining the target population and the challenges they face and providing crucial input into the design, delivery, and evaluation of the intervention [14].
- *Contextual determinants* assess the extent to which research teams consider factors that affect the implementation of the intervention, including barriers (hindrances) and facilitators (enablers) at various levels (systems, organizations, provider, and consumer/patient level). Eliciting determinants is vital to inform the selection of appropriate implementation and sustainment strategies.
- *Implementation and sustainment strategies* refer to how much research teams specify the supports and activities provided to individuals and organizations delivering the intervention to adopt and sustain the intervention. Implementation and sustainment strategies need to be documented and tracked for replicability and translation of study results and should address the identified contextual determinants.
- *Implementation and sustainment outcomes* consider whether research teams measure how well the intervention was implemented and the extent of its uptake by the intervention deliverers and receivers. Implementation and sustainment outcomes provide valuable data to enrich the assessment of patient health outcomes.

The initial measure was shared with implementation science expert colleagues, including the Methods & Measures Section of the Center for Dissemination & Implementation At Stanford (C-DIAS) Research Core. Their feedback informed refinements.

The version of the DIRC-SS piloted in the current study consisted of 15 items across the 5 focal dimensions (3 items per implementation science construct; see supplementary material). It was designed to be completed via self-assessment either by individual researchers or research teams without presumed exposure to or expertise in implementation research. Items were rated on a 5-point scale from *1* – *None*, *2 – Minimal/Some, 3 – Partial/Moderate, 4* – *Significant but not comprehensive,* to *5* – *Full/Comprehensive/Complete*. Raters select a 2 or 4 when they have not fully met 3 or 5, respectively. Dimension scores were calculated as a mean of the 3 ratings in that dimension, and the total score was calculated as the mean across all 15 items.

### Participants/settings

The information for this study was gathered from 10 research projects in the Helping End Addiction Long Term® Initiative (HEAL) Data2Action (HD2A) Program [15]. The HD2A Program, funded by the National Institute on Drug Abuse (NIDA), aims to integrate and apply existing data to enhance service delivery for the prevention and treatment of opioid use disorder (OUD) and pain. In addition to the research projects, the HD2A Program includes three support centers; two are focused on data infrastructure and on modeling and economic resources, respectively, and the third is the Research Adoption Support Center (RASC) [16]. The RASC plays a critical role by bringing implementation science expertise to the projects, guiding the selection and implementation of evidence-based practices to address service delivery needs, and evaluating the overall HD2A Program.

The HD2A Program’s 10 research projects were funded through two rounds of applications (6 in 2022, 4 in 2023). Each phased project includes a 1–2 year exploratory R61 for preparatory work, such as collecting pilot data, feasibility testing, and building infrastructure, and a 3–4 year developmental R33 for a larger study. The HD2A research projects are focused on one of two priorities: (1) improving data ecosystems (Acceleration Projects) or (2) addressing service delivery gaps with evidence-based strategies (Innovation Projects). Projects range from developing an overdose data dashboard that visualizes local, population-level data to help county-level overdose review teams identify opportunities for evidence-based prevention and treatment, to building local data infrastructure (e.g., emergency medical services reports, mobile integrated case management data, medical examiner data) to inform the creation of a sub-acute stabilization center for people at high-risk opioid overdose.

### Procedures

To provide a comprehensive assessment of the DIRC-SS, this study reports on results from both experts and non-experts. The 10 HD2A research teams were each matched with two implementation science experts from the RASC. Each research team and their RASC experts independently completed the time 1 (baseline) DIRC-SS within the first six months of funding. Research teams were encouraged to engage in group discussions and generate consensus ratings for each item based on their overall perceptions of the project’s current state. If teams were unable to reach consensus on a specific item, they were instructed to select the lowest rating suggested by team members and to provide comments. Once the research teams and RASC experts completed their respective ratings, they met virtually to share and discuss responses. Additionally, although not a required part of the DIRC-SS process, the research teams and RASC experts engaged in a collaborative process to identify pragmatic project-specific recommendations for dimensions with lower DIRC-SS scores. The RASC experts then provided ongoing support and consultation at an agreed-on cadence. At time 2 (one year later), the research teams and RASC experts each completed the DIRC-SS again, and either met virtually regarding ratings and ongoing support, or, in a few instances in which the research teams did not perceive ongoing support needs, exchanged emails. The Stanford University School of Medicine Institutional Review Board deemed this project “not research” as its primary purpose was program evaluation.

### Data analysis

Internal consistency was evaluated using Cronbach’s alpha for each dimension and the overall instrument [17]. Cohen’s Kappa statistic was used to assess inter-rater reliability. It is a quantitative measure of reliability for two raters who rate the same thing, correcting for how often the raters may agree by chance [18].

Changes in the DIRC-SS total score and five dimension scores over time were tested using Wilcoxon signed rank tests, along with associated effect sizes given the rank biserial correlation (*r*). Following guidelines proposed by Cohen [19], 0.10 was regarded as a small effect size, 0.30 a medium effect size, and 0.50 a large effect size.

Data were analyzed using the R Statistical Package [20]. There were very few missing values. One research team did not submit their DIRC-SS at time 1. There were no missing data at time 2.

## Results

### Scale reliability

The internal consistency reliability of the DIRC-SS was tested using time 1 data from the research teams and RASC experts. Table 1 shows Cronbach’s alpha coefficients for the total score and each dimension. Per standard guidelines for interpreting alpha coefficients [17], internal consistency reliability of the DIRC-SS total score was good. Reliability across the five dimensions was variable; two dimensions were in the acceptable range (Partner Engagement, Contextual Determinants), two in the questionable range (Evidence for the Intervention, Implementation and Sustainment Outcomes), and one in the poor range (Implementation and Sustainment Strategies).

**Table 1.** Reliability of DIRC-SS total score and dimensions across 10 research projects

Dimension	Meaning	Number of Items	Cronbach’s Alpha (*α*)
Total Score	Mean across items	15	0.80
Evidence for the Intervention	Intervention has evidence of efficacy and effectiveness	3	0.65
Partner Engagement	Representatives from the intervention site and community are involved in the intervention’s design, delivery, and evaluation	3	0.79
Contextual Determinants	Identification of barriers/facilitators to the intervention’s implementation	3	0.70
Implementation and Sustainment Strategies	Selection, adaptation and description of methods to support users with the intervention’s installation or sustainment	3	0.54
Implementation and Sustainment Outcomes	Measurement of how much and how well the intervention is delivered	3	0.66

Cronbach’s Alpha (α) interpretation: < 0.50 Unacceptable, 0.50–0.59 Poor, 0.60–0.69 Questionable, 0.70–0.79 Acceptable, 0.80–0.89 Good, > = 0.90 Excellent.

### Inter-rater reliability

At time 1, the mean kappa of research team and RASC expert ratings across items was 0.09, with a range of −0.21 to 0.80. Per Cohen’s original interpretation [18], this represents “none to slight agreement.” At time 2, the mean kappa was 0.40, with a range of −0.17 to 0.89, suggesting “moderate’ agreement.”

### Change in DIRC-SS ratings over time

**Figure 1.**
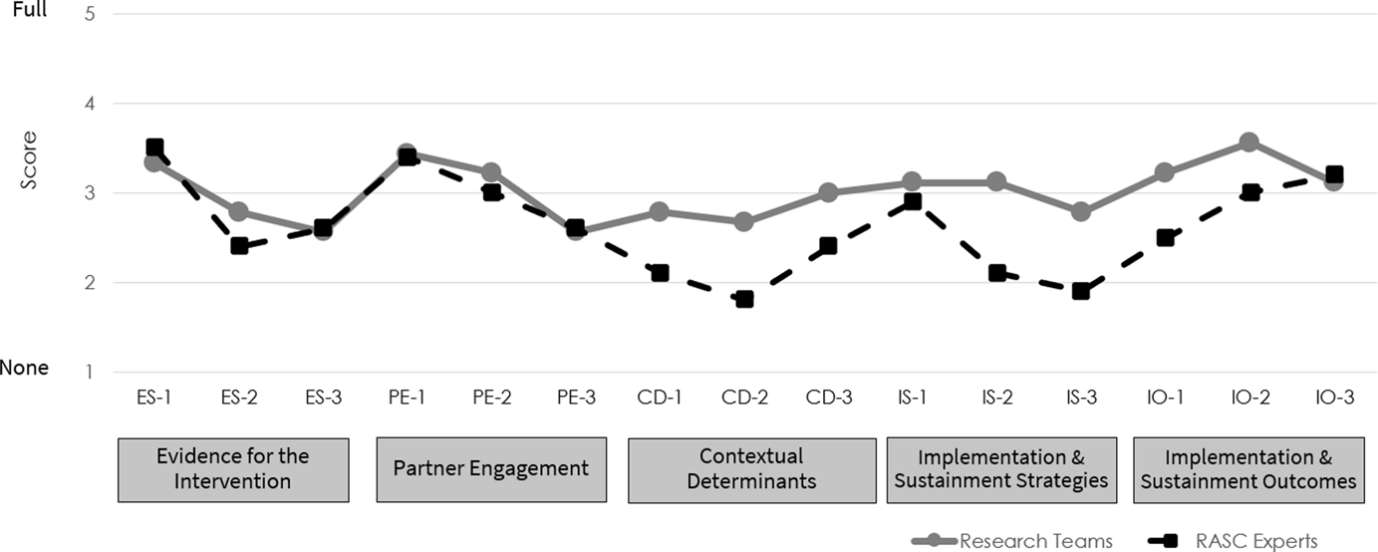
DIRC-SS item scores at time 1 rated by research teams (*n* = 9) and RASC experts (*n* = 10).

Figure 1 shows DIRC-SS item scores at time 1 as rated by the research teams and RASC experts. At baseline, most ratings were in the none to moderate range, suggesting, as expected, relatively little use of implementation research methods in these projects.

**Figure 2.**
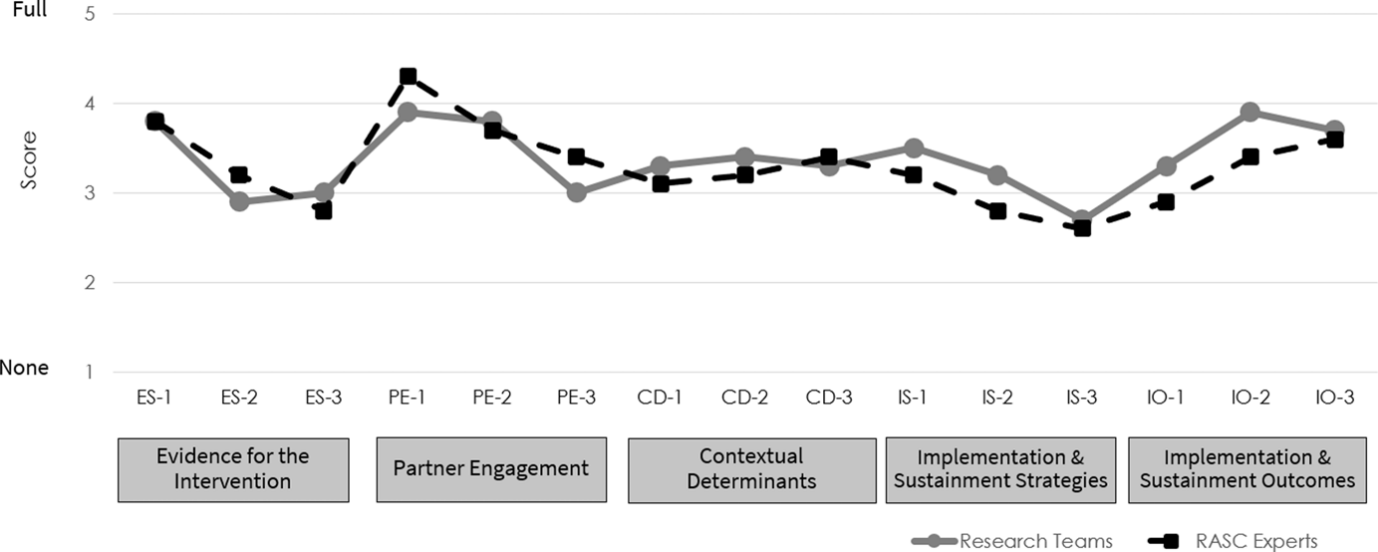
DIRC-SS item scores at time 2 rated by research teams (*n* = 10) and RASC experts (*n* = 10).

Figure 2 shows DIRC-SS item scores at time 2. Time 2 ratings generally fell in the moderate to full range, indicating substantial progress in including implementation methods in the research projects. The mean total score for the research teams (3.73) was slightly higher than the RASC experts (3.63; not reported in Figures or Tables). The largest discrepancy between ratings occurred for the Implementation and Sustainment Outcomes item IO-2 (having plans to measure implementation outcomes; see Appendix 1 for full item), with a difference of 1.0 point between research team and RASC expert assessments. Research team and RASC expert ratings were identical or nearly identical for several items, including Partner Engagement items PE-2 and PE-3 (items regarding partners from the community or underrepresented groups, respectively, having input in planning the project), Implementation and Sustainment Strategies item IS-3 (having plans to track the delivery of implementation strategies), and Implementation and Sustainment Outcomes item IO-3 (meeting with partners regarding sustainment).

Table 2 shows change in DIRC-SS over time for each dimension for the research teams and the RASC experts, including the mean difference, results of Wilcoxon Signed Ranks test, *p* values, and corresponding effect sizes. For the research team ratings, the changes in total score and dimension scores did not significantly change over time, except for a significant increase in Partner Engagement. However, for the RASC expert ratings, the total score and the Partner Engagement, Contextual Determinants, and Implementation and Sustainment Strategies dimensions showed statistically significant increases. Given the small sample size and low statistical power of this study, the effect sizes shed light on the magnitude and direction of change. Per Cohen’s [19] guidelines, all changes over time were large effect sizes, except for the change in research team ratings for Implementation and Sustainment Strategies, which had a medium effect size.

**Table 2.** Changes in research team and RASC expert ratings of the DIRC-SS from time 1 to time 2 (*n* = 10) with results of Wilcoxon Signed Ranks Tests and effect sizes

	Research team ratings				RASC expert ratings			
Variable	Mean difference	Wilcoxon *W*	*p*	Effect size *r*	Mean difference	Wilcoxon *W*	*p*	Effect Size *r*
Total score	0.25	4.5	0.207	0.71	0.67	3	0.012	0.79
Evidence for the intervention	0.22	3	0.115	0.77	0.43	3	0.062	0.79
Partner engagement	0.41	0	0.040	0.89	0.80	0	0.018	0.89
Contextual determinants	0.44	6	0.175	0.65	1.13	0	0.012	0.89
Implementation and sustainment strategies	−0.04	10.5	1.000	0.47	0.57	4	0.028	0.76
Implementation and sustainment outcomes	0.22	2	0.273	0.81	0.40	6	0.175	0.69

Wilcoxon Signed Ranks Test (W) is a non-parametric test of a significant difference between two paired samples. Effect size (*r*) interpretation: 0.10–0.29 small, 0.30–0.40 medium, > = 0.50 large.

## Discussion

The DIRC-SS is the first instrument to assess the use of implementation research methods in non-implementation focused research projects in a standardized, transparent, generalizable way. Unlike the INSPECT and StaRI tools, the DIRC-SS focuses on five key implementation science constructs and can be completed at any point in the research process. While the Implementation Research Logic Model contains four of these constructs and is extremely useful in planning elements of a study [13], it does not provide the opportunity for scoring and examining the presence of key implementation science constructs across a group of studies. The DIRC-SS is a brief, 15-item scale that can be completed by a research team or expert raters, and it has applicability for both research and non-research projects, particularly in the Clinical Research stage.

This study was an initial exploration of the DIRC-SS in 10 NIH-funded research projects that were not required to use implementation science in their applications. Results indicate that the DIRC-SS total score showed good internal consistency. Inter-rater reliability between research teams and RASC experts began low but increased from baseline to one-year follow-up, from slight to moderate agreement. These projects received information and education on implementation science from the RASC, so the increase in internal consistency may be due to the research teams having a better understanding of implementation research methods and ability to accurately rate inclusion of these principles in their projects.

The study also described change in DIRC-SS ratings from baseline to one-year follow-up. For the RASC expert ratings, the total score and scores within three dimensions showed significant increases over time, and effect sizes across almost all research team and RASC expert ratings suggested large effects, even in this small sample. These changes reflect how several of the research projects began to include implementation research methods in their work and highlight the utility of the DIRC-SS to inform the provision of targeted implementation science support.

The development of the DIRC-SS is timely, given recent recommendations of the cross-domain working group of the CTSA consortium to integrate implementation science into all CTSAs and to develop “common and pragmatic measures…to track…the application of implementation science principles into research practice” [21, p. 3]. The DIRC-SS is a pragmatic tool that can be used by researchers and intervention developers in the Clinical Research stage to augment their work.

DIRC-SS findings can be used to examine implementation research capability at the individual project level or at the institutional level. At the individual project level, the DIRC-SS can help to guide research teams on how to rigorously integrate implementation research concepts and methods into their grant applications and ongoing projects. Considering implementability early in the intervention development or evaluation process can increase the chances of effective translation downstream and maximize public health benefit. To facilitate consultations with individual projects, the DIRC-SS can be used as a consultation or capacity building tool [7,22], including as the cornerstone of the CTSA consultation process. In this study, results from the DIRC-SS were shared with each of the individual HD2A projects and used as part of a shared decision-making process to determine what implementation support might be needed. The DIRC-SS findings stimulated ideas about potential pragmatic opportunities to enhance implementation research methods in each project, such as using community based participatory research principles to more fully engage community partners in the research process and build buy-in for an intervention, or adding a measure of contextual determinants to better understand barriers and facilitators to the intervention’s adoption that may inform what supports are needed to engender the interventions’ uptake. An ongoing formative evaluation of the HD2A network revealed that most projects found consultation from the RASC to be helpful (Palinkas et al., unpublished). In contrast to other project-specific tools, the DIRC-SS can also be used mid-project, allowing the possibility for bolstering implementation methods midstream, such as improved tracking implementation strategies across sites, or at the end of the project, to identify the need for an additional measure of implementation outcomes (e.g., adoption of the intervention by providers, teams, or clinics).

At the institutional level, aggregating DIRC-SS data across multiple research teams’ grant portfolios, by department, career stage, grant mechanisms, and over time, would provide valuable information about the development of implementation research capability within a CTSA. A CTSA hub, for example, could embed the DIRC-SS either as a self-survey or conducted by objective expert raters, with an online version to facilitate tracking and reporting. These data could provide the CTSA with objective information to identify high performers, signal training/education needs, and evaluate the impact of consultation, training, and education in raising the implementation science bar across projects at their institution [21,23]. Similarly, a funding agency could use DIRC-SS results to understand future implementability of developed interventions, plan support to a group of projects as in the HD2A program, develop language for funding opportunities around embedding implementation research methods into applications, or create new funding opportunities that address implementation research gaps [24].

The DIRC-SS can also be used outside of the NIH-funded research context in community-based or system-level implementation initiatives. For example, philanthropic funders are increasingly interested in pilot or demonstration projects that can show high-impact in real-world settings [25]. The DIRC-SS documents the use of implementation constructs that can increase the likelihood that interventions are scaled up and out. Moreover, the DIRC-SS can be used in single practice improvement projects or across a group of projects (e.g., funding by a health system in multiple locations).

### Limitations

Several limitations of this study need to be noted. One limitation is the small sample: 10 research projects within one NIH-funded program focused on substance use and pain management. Notably, however, these studies do encompass a variety of interventions, contexts, and research designs. Generalizability of the DIRC-SS should be examined further across a broader range of studies and clinical focus areas, as well as stage of development (e.g., concept development, proposal development, funded study execution). Yet, there is reason to think that the findings would carry through to other health concerns. Another possible limitation is that the internal consistency of several of the DIRC-SS dimensions was questionable or poor. However, items can be valuable even if they are not inter-correlated within a scale, and we may not expect items within a specific implementation science domain to be internally consistent if they reflect different levels of implementation research acumen; for instance, in the implementation strategies dimension, determining how to implement an intervention is a beginner-level concept, whereas matching strategies to barriers and facilitators is a more intermediate- to advanced-level concept [22]. In addition, although the scale was designed as a self-survey, the low initial level of inter-rater reliability between the research teams and RASC experts at baseline suggests that some knowledge of implementation science principles may be needed to accurately complete the scale. These differences suggest an opportunity to educate and consult with the research team, as through shared decision-making, to consider enhancements to the proposal or project. The fact that agreement improved over time, following information and support from the implementation science support center (RASC), is an indication and validation of this reasoning. However, to further address this limitation and enhance standardization across research teams and consultation programs, we are developing a user-guide that provides brief definitions, examples, and key considerations for each item. This will support more consistent and reliable application of the DIRC-SS across research teams and CTSA hubs.

### Conclusion

To further move translational science forward, intervention researchers need to move beyond effectiveness research accompanied by superficial implementation preparation to proactive, substantive implementation research whereby implementation methods are integrated even before effectiveness has been established [26]. Previous reviews of NIH-funded projects have shown a relative lack of emphasis on the measurement of contextual determinants, specification of implementation strategies used, and information on implementation outcomes [27,28]. The DIRC-SS can provide clear avenues to help push research teams and funders to more sophisticated implementation research in the Clinical Research stage that can help realize the benefits of translational science.

## Supporting information

10.1017/cts.2026.10711.sm001 Gotham et al. supplementary material Gotham et al. supplementary material

## Supplementary material

The supplementary material for this article can be found at https://doi.org/10.1017/cts.2026.10711.
