# Supplementary material for: A tool to evaluate the integration of implementation science methods in a research project: The Dissemination and Implementation Research Capability Self Survey (DIRC-SS)
Source: J Clin Transl Sci. 2026 Feb 13;10(1):e55. doi: 10.1017/cts.2026.10711 (PMC13040393; doi:10.1017/cts.2026.10711)
Supplement: Gotham et al. supplementary material [file S2059866126107110sup001.docx]

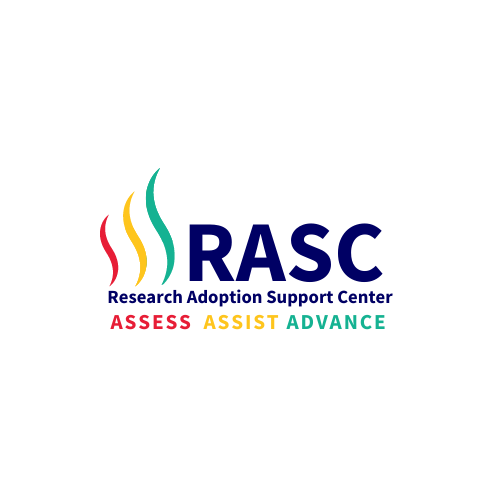
**
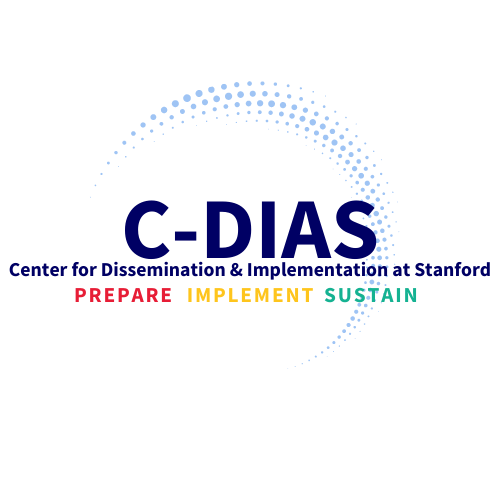
**

**Dissemination and Implementation**

**Research Capability – Self Survey**

**(DIRC-SS)**

**Version 1.4, 1.29.2024**

This work was supported by the National Institute on Drug Abuse (NIDA), National Institutes of Health (NIH) under award numbers P50DA054072 (PI: McGovern) and U2CDA057717 (Contact PI: McGovern). The content is solely the responsibility of the authors and does not represent the official position of NIDA/NIH.

McGovern, M. P., Chokron Garneau, H., Gotham, H. J., Becker, S., Becker, W., & Brown, C. H. (2024). Dissemination and Implementation Research Capability – Self Survey (DIRC-SS). Center for Dissemination and Implementation at Stanford (C-DIAS) and HEAL Data2Action Research Adoption Support Center.

**Dissemination and Implementation Research Capability – Self Survey**

**(DIRC-SS)**

**Purpose**

The purpose of the DIRC-SS is to gather systematic information from an existing or proposed research project about the current state and stimulate ideas about potential pragmatic opportunities to enhance the D&I science aspects to the project. The overarching goal is to consider “implementability” early in the intervention development process to increase the chances of effective translation downstream.

**Instructions**

The **Dissemination & Implementation Research Capability (DIRC) Self Survey (SS)** is a research team-based appraisal of the degree to which a project incorporates key components of D&I methods. Together with your key research team members (PI, MPI, Co-PI, Co-I, project directors/coordinators), please respond to the following questions based on a group discussion. It is best to develop a consensus rating response where possible. For each question, you can provide details and/or examples from your project that led to your rating. In instances where there is disagreement about a rating, it is generally best to go with the lower rating. If consensus cannot be reached, or if a question does not appear relevant, please elaborate in the comment section of each dimension.

**Project Summary**

1. **Title:**
2. **PI(s):**
3. **Population/Patient Problem:**
4. **Intervention/program/service(s) being developed/evaluated/implemented:**
5. **Comparator:**
6. **Type of setting(s):**
7. **Type of person(s) delivering the intervention:**

**7a. Research personnel, type:**

**7b. Existing staff members, type*:***

1. **Who participated in completing the DIRC-SS (list all):**
2. **Time spent completing the DIRC-SS:**

**Orienting Your Team to the DIRC-SS**

There are 5 key dimensions in the DIRC-SS:

1. ***Evidentiary Support*** for the Intervention/Program/Service;
2. Planning for ***Partner Engagement*** to help ensure that interventions will be effective across diverse groups/contexts and used and sustained in practice over time;
3. Consideration of ***Contextual Determinants*** that may impact the reach and adoption of the intervention/program/service;
4. Selection, adaptation and description of ***Implementation and Sustainment Strategies***, operationalized as the steps and methods taken to support users (within the project or in the real world) with the installation or sustainment of the intervention/program/service
5. Evaluation of ***Implementation and Sustainment Outcomes***, which are the effects of actions to implement or sustain the intervention/program/service (how much and how well an intervention was implemented/sustained).

These 5 dimensions each include 3 benchmark items, yielding ***15 DIRC-SS items total***.

Use this consensus rating scale for each item to capture your team’s overall perception of the project (as is or as proposed):

**1: None**

**2: Minimal/some**

**3: Partial/moderate**

**4: Significant but not complete**

**5: Full/comprehensive /complete**

Use the **Description** section provide details or propose questions.

| **1. Evidentiary support for the intervention/program/service** | | |
| --- | --- | --- |
| **Key Component/Activity** | **Rating** | **Description** |
| 1. The intervention itself. |  |  |
| 1. Our approach to delivering the intervention, the intervention delivery platform. |  |  |
| 1. Our adaptation of the intervention or delivery approach (for increased effectiveness or accessibility). |  |  |
| Comments: | | |

| **2. Partner Engagement** | | |
| --- | --- | --- |
| **Key Component/Activity** | **Rating** | **Description** |
| 1. Key and representative partners from the project site(s) had input into defining the population, clinical problem, or intervention/program/service being delivered. |  |  |
| 1. Key and representative partners from the community in which the project takes place had input into defining the population, clinical problem, or intervention/program/service being delivered. |  |  |
| C. If persons from historically underrepresented groups are potential participants in the project, key partners from these groups had input into defining the population, clinical problem, or intervention/program/service being delivered. |  |  |
| Comments: | | |

| **3. Contextual Determinants (systems, organizational, provider, and patient/consumer-level factors that affect implementation of the intervention, i.e., barriers and facilitators)** | | |
| --- | --- | --- |
| **Key Component/Activity** | **Rating** | **Description** |
| A. We have methods to assess the barriers to implementing our intervention/program/service that may exist at the systems, networks, policy, financing/reimbursement, community, and/or cultural level. |  |  |
| B. We have methods to assess the barriers to implementing our intervention/program/service in our study site organizations—such as factors related to leadership, workflow, workforce, readiness, resources, and overall fit with patients (or consumers). |  |  |
| C. We have methods to assess any necessary modifications to our intervention/program/service to increase the likelihood of implementation. |  |  |
| Comments: | | |

| **4. Implementation and Sustainment Strategies (processes, methods, activities, and resources that support implementation and sustainment of the project intervention/program/service, e.g., training, facilitation/coaching, incentives, performance data, audit and feedback)** | | |
| --- | --- | --- |
| **Key Component/Activity** | **Rating** | **Description** |
| A. Related to items 2A-C above, we have met with key partners for guidance about the best ways (the “HOW”) to implement our intervention/program/service (the “IT”). |  |  |
| B. We are selecting implementation strategies to install our intervention based on some of the barriers that we may encounter—in order to address or circumvent these barriers, or based upon known evidence for effectiveness of these implementation strategies. |  |  |
| C. We have detailed plans to track the participation, delivery, procedures, adaptations, fidelity, and costs of the implementation strategies we are using to install the intervention/program/service. |  |  |
| Comments: | | |

| **5. Implementation and Sustainment Outcomes, assessing the effects of actions to implement the intervention/program/service including extent of use by the deliverers (adoption) and receivers (reach)** | | |
| --- | --- | --- |
| **Key Component/Activity** | **Rating** | **Description** |
| A. In addition to patient level outcome measures, we have plans to evaluate implementation outcomes such as acceptability, feasibility, and appropriateness of the intervention/program/service. |  |  |
| B. In addition to patient level outcome measures, we have plans to evaluate implementation outcomes such as: Reach (proportion of patients who receive the intervention of all eligible), Adoption (proportion of intervention deliverers of all eligible), Implementation Fidelity (adherence to guidelines for intervention as designed), and Equity (analyses of differential participation, engagement and response based on social determinant factors). |  |  |
| C. We have met with relevant policymakers or payers who may inform the sustainability of the intervention/program/service. |  |  |
| Comments: | | |
